# Clinical Utility of Circulating Tumor Cells in *ALK*-Positive Non-Small-Cell Lung Cancer

**DOI:** 10.3389/fonc.2014.00281

**Published:** 2014-11-05

**Authors:** Vincent Faugeroux, Emma Pailler, Nathalie Auger, Melissa Taylor, Françoise Farace

**Affiliations:** ^1^INSERM U981 “Identification of Molecular Predictors and New Targets for Cancer Treatment,” Institut Gustave Roussy, University of Paris-Sud, Paris, France; ^2^Translational Research Laboratory, Institut Gustave Roussy, Paris, France; ^3^Department of Biopathology, Institut Gustave Roussy, Paris, France

**Keywords:** *ALK* rearrangement, circulating tumor cells, targeted therapy, non-small-cell lung cancer, predictive biomarker

## Abstract

The advent of rationally targeted therapies such as small-molecule tyrosine kinase inhibitors (TKIs) has considerably transformed the therapeutic management of a subset of patients with non-small-cell lung cancer (NSCLC) harboring defined molecular abnormalities. When such genetic molecular alterations are detected the use of specific TKI has demonstrated better results (overall response rate, progression free survival) compared to systemic therapy. However, the detection of such molecular abnormalities is complicated by the difficulty in obtaining sufficient tumor material, in terms of quantity and quality, from a biopsy. Here, we described how circulating tumor cells (CTCs) can have a clinical utility in anaplastic lymphoma kinase (*ALK*) positive NSCLC patients to diagnose *ALK-EML4* gene rearrangement and to guide therapeutic management of these patients. The ability to detect genetic abnormalities such *ALK* rearrangement in CTCs shows that these cells could offer new perspectives both for the diagnosis and the monitoring of *ALK*-positive patients eligible for treatment with ALK inhibitors.

In the past decade, the treatment of non-small-cell lung cancer (NSCLC) has considerably shifted with the emergence of rationally targeted therapies for a subset of molecularly defined lung cancers. NSCLCs and in particular adenocarcinoma, the most frequent histologic subtype, have been segmented into clinically relevant molecular subsets according to a classification based on multiple so-called oncogenic “driver” alterations (Figure 1A) (1). These somatic aberrations occur in genes that encode signaling proteins crucial for tumor proliferation and survival. Tumors harboring these mutant oncogenes may be systematically identified and targeted specifically using tyrosine kinase inhibitor (TKI) therapies that ensure dramatic and durable clinical benefit. The first example of a clinically relevant NSCLC driver oncogene was the identification of somatic mutations in the epidermal growth factor receptor (*EGFR*) gene (2–4).Common *EGFR* alterations (the L858R point mutation and exon 19 deletions) are present in 10–30% of patients with NSCLC and confer sensitivity to gefitinib, erlotinib, and afatinib. As first-line treatment, EGFR inhibitors can produce overall response rates (ORR) of 75% in selected NSCLC patients (5).

**Figure 1:**
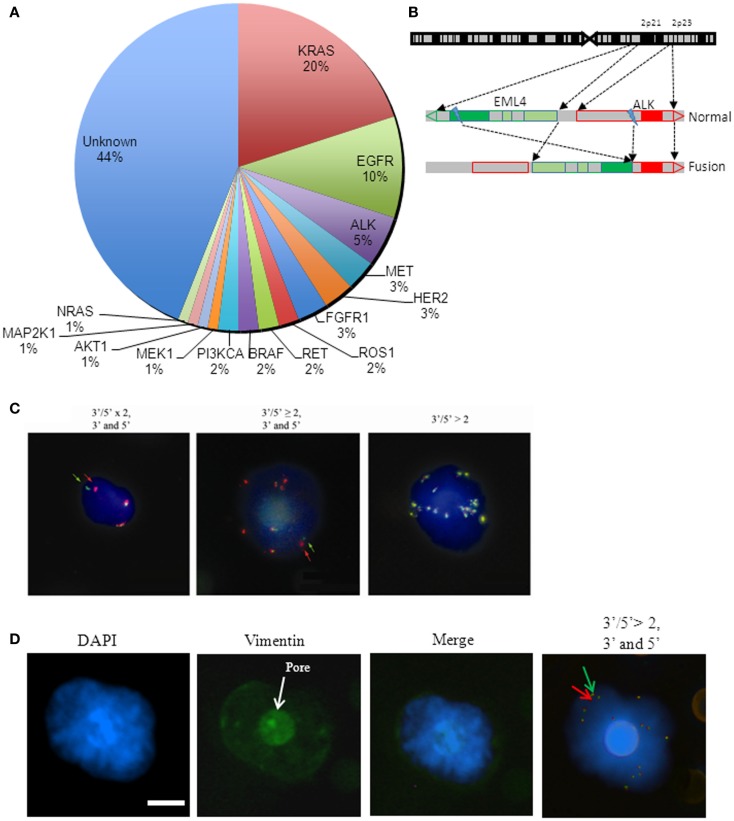
**Molecular characterization of CTC in *ALK*-rearranged non-small-cell lung cancer**. **(A)** Pie chart of NSCLC mutations and scheme representation of *ALK* rearrangement. Percentage of known somatic mutations in oncogenic drivers in NSCLC. Pie chart of NSCLC mutations. **(B)** Scheme of *EML4-ALK* rearrangement. The two genes are located on chromosome 2 in position 2p21 and 2p23, respectively for *EML4* and *ALK*. The rearrangement is the result of a chromosomal inversion. **(C)** Examples of *ALK*-gene abnormalities detected by FA-FISH. *ALK*-rearranged CTC with an *ALK* native copy (yellow signal) and an *ALK-*rearranged copy. Arrows show the *ALK* rearrangement with a split 3′ and 5′ (red/green) signal (left). *ALK*-rearranged CTC with a gain of native *ALK* copies (yellow signals) and an *ALK*-rearranged copy as shown in **(A)** and indicated by arrows (middle). CTC with a gain of *ALK* native copies (right). **(D)** Example of *ALK-*rearranged mesenchymal CTC. Immunofluorescence staining (Hoechst/Vimentin) and *ALK*-rearranged CTC with gain of *ALK* native copy (yellow signals) and *ALK*-rearranged copy. Arrows show the *ALK* rearrangement with a split 3′ and 5′ (red/green) signal. Scale bar: 8 μm.

Similarly to *EGFR* mutations, the *ALK*-gene rearrangement defines a unique molecular subset in 3–7% of NSCLC patients (6). *ALK*-gene rearrangement involves the anaplastic lymphoma kinase (*ALK*) gene and, most often, the echinoderm microtubule-associated protein-like 4 (*EML4*) loci which both map to the short arm of the human chromosome 2 in opposite directions (7, 8) (Figure 1B). Several different in-frame fusion variants of *EML4-ALK* have been described with different *EML4* breakpoints. However, all fusion variants contain the same C-terminal domain, which confers a gain of function resulting in constitutively active fusion proteins with potent transforming activity. The clinical characteristics of NSCLC patients that are positive for *EML4-ALK* variants are similar to those of who harbor activating mutations in the *EGFR* gene: both groups of patients tend to manifest an adenocarcinoma histological subtype and to be non or light smokers (9). The discovery that the EML4-ALK fusion protein was a potent oncogenic driver in NSCLC rapidly fueled the development of the multi-targeted TKI crizotinib, and accelerated its FDA approval for the treatment of patients with advanced *ALK*-positive NSCLC. Two studies have since shown an ORR of 60% and a median progression free survival (PFS) of 8–9 months in *ALK*-positive NSCLC patients receiving crizotinib (10). Another study compared treatment with crizotinib to chemotherapy in ALK-positive NSCLC patients and showed that the PFS was longer for patients receiving crizotinib (7.7 months) than for those treated with chemotherapy (3 months) (11). Despite this high response rate in *ALK*-rearranged NSCLC, most patients develop resistance to crizotinib, typically within 1 year. Next generation ALK inhibitors with potentially improved efficacy and selectivity compared to crizotinib are currently being developed to overcome this resistance to crizotinib. In a recent phase I study, ceritinib demonstrated marked anti-tumor activity in both crizotinib-relapsed and crizotinib-naive patients (12).

Prospective molecular profiling of NSCLC is now performed in routine clinical practice to identify oncogenic “driver” abnormalities and to screen eligible patients for targeted TKI therapies. Tumor tissue may be difficult to obtain in advanced/metastatic NSCLC patients for whom surgery is rarely a component of the treatment strategy. The molecular characteristics or biomarkers are most commonly identified on tumor biopsy samples although tissue adequacy, both in terms of quantity and quality, is often insufficient for patients with advanced/metastatic NSCLC. The detection of an *ALK* rearrangement is currently performed on small biopsies or fine-needle aspirates of the tumor but is hindered by the very limited tissue quantities available. The diagnosis of *ALK* rearrangement can be performed by fluorescence *in situ* hybridization (FISH, which is considered as the gold standard), immunohistochemistry (IHC), or reverse transcriptase-polymerase chain reaction (RT-PCR) on these tumor samples. Wang et al. recently reported that FISH and IHC results were concordant in 98% of cases while RT-PCR results were less concordant with FISH (89%). FISH is highly specific but is costly and requires technical expertise. The IHC assay developed by Ventana (Ventana ALK assay) enables to detect *ALK* rearrangement with a high sensitivity and concordance when compared to FISH results, and has the advantage to be automated, faster, and less expensive than FISH (13). Treatment with crizotinib was FDA approved with a companion diagnostic test, the Vysis *ALK* break apart FISH probe kit (Abott molecular). Finding alternatives to a tumor biopsy and more effective means to diagnose an *ALK* rearrangement is a critical issue in order to identify NSCLC patients who may benefit from an ALK inhibitor treatment.

Molecular characterization of circulating tumor cells (CTCs) may inform on the status of predictive biomarkers for drug sensitivity and therapy selection. CTCs are, however, very rare events occurring at rates, as low as one cell per 10^6^ or 10^7^ leukocytes. Most methods of CTC detection rely on the combination of two successive steps, an initial enrichment process followed by CTC detection so as to increase the sensitivity of the assay (14). Numerous technical efforts have been made to reliably detect and quantify CTCs, although the development of a universal assay has proven quite difficult. The major technical challenges for CTC detection are due to the rarity of CTCs and their high degree of phenotypical and molecular heterogeneity. Using the CellSearch platform, which is based on the detection of epithelial cells expressing EpCAM, CTCs levels have been observed to be prognostic in various metastatic solid tumors including NSCLC and SCLC. Using an enrichment technique based on blood filtration (ISET, isolation by size epithelial tumor cells), the prognostic value of CTCs was also reported in patients with resected NSCLC. We and others groups have reported that CTCs are identified in higher numbers using the ISET technique compared to the CellSearch method in NSCLC most likely due to the fact that CTCs expressing markers of epithelial–mesenchymal transition (EMT) and that have lost epithelial features are missed by CellSearch (15). In spite of technical difficulties, a few studies have demonstrated the feasibility of CTC assays for predictive biomarker detection. Two groups including our own have reported the feasibility of detecting *ALK* rearrangement from CTCs enriched by filtration in patients with *ALK*-positive NSCLC (16, 17). Ilie et al. reported the detection of *ALK* rearrangement and strong ALK protein expression by IHC in CTCs from five patients with *ALK*-rearranged NSCLC. FISH and IHC were negative in CTCs from 82 NSCLC patients whose tumors did not harbor *ALK* rearrangement. In order to exploit CTCs as predictive biomarkers of personalized treatments, our group developed a FISH method on filters (filter adapted-FISH, FA-FISH) that was optimized for high cell recovery. By combining blood filtration and FA-FISH, we demonstrated that *ALK*-rearranged CTCs could be detected in a cohort of 18 *ALK*-positive NSCLC patients (17). All 18 *ALK*-positive patients had four or more *ALK*-rearranged CTCs per milliliter of blood while no or only one *ALK*-rearranged CTC was detected in 14 *ALK*-negative patients (Table 1). Furthermore, all CTCs harbored a unique *ALK-*rearrangement pattern consisting in the 3′5′ break apart of *ALK* probes whereas heterogeneous rearrangement patterns were present within the tumor. This unique 3′5′ pattern was present in CTCs harboring either a single copy of *ALK* or a gain of native *ALK* copies (Figure 1C). CTCs harboring an isolated red signal pattern were never detected in *ALK-*positive patients, even when isolated red signals were exclusively present in the tumor biopsies. The split rearrangement pattern was therefore detected in CTCs regardless of the frequency of tumor cells harboring this rearrangement in the tumor tissue. *ALK*-rearranged CTCs harboring this unique rearrangement expressed a mesenchymal phenotype, suggesting that these cells may have derived from the clonal selection of tumor cells harboring greater invasive and migratory properties (Figure 1D). In this study, we also demonstrated that monitoring quantitative and qualitative changes of CTCs with distinct *ALK* abnormalities pattern was feasible in patients undergoing ALK inhibitor therapy.

**Table 1:** **Numbers and percentages of ALK-rearranged cells in tumor and in CTCs of ALK-positive and ALK-negative patients**.

ALK positive (P) or negative (PN) patients^a^	Sex	Age	Smoking status^b^	Tumor % of rearranged cells^c^	CTCs characterized by ISET		
					Total CTCs (/mL)^d^	Rearranged CTCs (/1 mL)	% of rearranged CTCs^e^
P1	M	32	3	97%	16	9	56%
P2	M	35	0	47%	17	9	53%
P3	F	40	0	30%	10	5	50%
P4	M	54	0	30%	9	4	44%
P5	F	79	0	60%	25	10	40%
P6	F	69	0	43%	28^g^	34	100%
P7	M	69	20	27%	25	7	28%
P8	M	48	3.5	RT-PCR+^f^	25	24	96%
P9	F	70	40	61%	15	6	40%
P10	M	53	0	30%	17	7	41%
P11	F	25	0	68%	16	7	44%
P12	F	44	12.5	29%	10	9	90%
P13	F	36	0	77%	7	4	57%
P14	M	48	5	62%	17	11	65%
P15	F	42	0	25%	10	7	70%
P16	F	52	0	26%	14	9	64%
P17	F	42	10	25%	10^g^	11	100%
P18	F	57	0	28%	18^g^	25	100%
PN1	F	42	0	0%	45	0	0%
PN2	M	60	60	0%	26	0	0%
PN3	M	67	35	0%	4	1	0%
PN4	F	57	35	NA	42	1	2%
PN5	M	76	52	NA	13	0	0%
PN6	F	53	20	IHC	20	1	5%
PN7	F	53	45	NA	33	1	3%
PN8	M	44	40	NA	37	0	0%
PN9	M	54	30	0%	20	1	5%
PN10	M	59	40	0%	19	1	5%
PN11	F	59	20	0%	10	1	10%
PN12	M	66	100	0%	23	1	4%
PN13	M	66	75	0%	12	1	8%
PN14	M	75	60	0%	16	1	6%

*ALK, anaplastic lymphoma kinase; CTC, circulating tumor cell; FISH, fluorescence *in situ* hybridization; NA, not available; RT-PCR, reverse transcription polymerase chain reaction*.

*^a^All patients have metastatic disease*.

*^b^Number of pack-years. All patients are former or never smokers (0: never smokers)*.

*^c^Percentage of rearranged tumor cells determined by FISH in tumor samples*.

*^d^Total numbers of CTCs per milliliter were calculated as the mean of CTCs identified by combining a four-color immunofluorescent staining with cytomorphological examination in 3 × 1 mL of blood*.

*^e^Percentage of ALK-rearranged CTCs is the proportion of ALK-rearranged CTCs determined by FA-FISH among total numbers of CTCs determined in independent experiments by combining a four-color immunofluorescent staining with cytomorphological examination*.

*^f^The biopsy was negative by FISH but positive by RT-PCR*.

*^g^In this three patients, numbers of ALK-rearranged CTCs were slightly greater to the number of total CTCs identified by phenotypic analysis. This difference is due to the fact that numbers of CTCs may differ between each spot of the filter*.

By demonstrating that *ALK* rearrangement can be reliably detected in the CTCs of *ALK*-positive NSCLC patients, these two studies provide new perspectives for the diagnosis of *ALK*-positive patients eligible for treatment with ALK inhibitors. These non-invasive molecular analyses performed on CTCs could be easily repeated at different time-points during treatment and could help to guide therapeutic decisions in a patient’s treatment course. Single cell analyses of CTCs captured on filters at the time of disease progression are also anticipated to help characterize resistant subclones and mechanisms of resistance to ALK inhibitors.

## Conflict of Interest Statement

The authors declare that the research was conducted in the absence of any commercial or financial relationships that could be construed as a potential conflict of interest.
